# Analysis of Dose Distribution in the Heart for Radiosurgical Ablation of Atrial Fibrillation

**DOI:** 10.7759/cureus.703

**Published:** 2016-07-18

**Authors:** Edward A Gardner, Georg A. Weidlich

**Affiliations:** 1 R&D, CyberHeart Inc.; 2 Radiation Oncology, Community Regional Medical Center

**Keywords:** cyberknife radiosurgery, stereotactic arrhythmia radioablation, treatment planning, electrophysiology, radiosurgery, arrhythmia

## Abstract

In a treatment planning study, radiosurgical treatment plans designed to produce lesions on the left atrium were created using two different methodologies. In one, structures in the heart (mitral valve and coronary arteries) were designated as critical structures while this was not done in the second plan. The treatment plans that were created were compared with standards for heart dose used when treating spine tumors. Although the dosage for the whole heart greatly exceeded the dose standards, when only the dose to the ventricles was considered, the plan where the mitral valve was spared was very close to the dose standards. The ventricles received a substantially higher dose in the plan where the mitral valve was not a critical structure. Although neither treatment plan was delivered, this study demonstrated the feasibility of treating the heart while minimizing dose to the ventricles.

## Introduction

CyberHeart (CyberHeart Inc., Mountain View, CA) has developed a method where radiosurgery is used to create a lesion in the heart. The scar tissue in this lesion blocks the transmission of electrical impulses in the heart in the same way that scar produced by Radio Frequency (RF) ablation isolates aberrant signals. Tests of this technique have included phantom studies and animal studies [1-3]. This method has been used in humans for treatment of both ventricular tachycardia [4] and atrial fibrillation [5]. 

In thoracic radiosurgery, the heart is considered as a critical structure and the dose it receives is carefully limited. Radiation exposure of the heart has been shown to produce risks such as accelerated coronary artery disease in radiotherapy patients. In particular, patients treated for Hodgkin’s disease (large volumes of the heart treated, large doses) with wide field radiation to the chest showed increased risk of cardiac disease in the decades following their treatment. If radiosurgery is to be used to treat cardiac arrhythmias, the danger to the heart caused by the radiosurgical treatment must be assessed. This paper reports on a treatment planning study where different methodologies were used. The feasibility of this approach has been presented at the International Society of Stereotactic Radiosurgery meeting, Paris-May 2011, entitled: “3-D Cardiac Contouring and Treatment Planning for Cardiac Ablation of Atrial Fibrillation (CyberHeart).”

Treatment plans created to produce pulmonary vein isolation in two different patients were analyzed to determine the distribution of radiation dose outside of the target. Neither of these plans was delivered clinically. The two treatment plans exemplify two different planning strategies and allow the relative merits of these strategies to be compared. This report summarizes this analysis.

## Technical report

### Treatment plans

Two treatment plans were analyzed from two separate patients. The plans were created from anonymized CT data from hospitals collaborating with CyberHeart. The plans were created for the CyberKnife (Accuray, Inc. Mountain View, CA.) system using MultiPlan 2.1™ (Accuray, Inc.) treatment planning software. Plan one was designed to isolate the pulmonary veins using a hybrid box/wide-area circumferential ablation (WACA) lesion set where a box lesion is used along with a line between encircling the right pulmonary vein pair. Plan two used a box lesion without an additional target on the posterior wall of the left atrium.

These lesion sets were chosen to avoid the esophagus that typically is positioned very close to either the left or right pulmonary veins. The box design provides ablation lesions that would create an electrical block from the pulmonary veins to the anterior left atrium and the ventricles while avoiding the esophagus. Adding an ablation line around one pair of pulmonary veins provides additional scar to stop electrical signal propagation.

In both plans, optimization considered dose to the target and non-cardiac critical structures: esophagus, bronchi, and skin. Tuning structures were utilized in both plans to confine the dose near the target region. A key difference between the plans was that Plan one was optimized to spare the mitral valve annulus as well as the circumflex and right coronary arteries while no critical structures were defined in the heart for Plan two. 

Both plans used a 25 Gy prescription dose. Table 1 lists characteristics that describe the plans. Because of the target shapes and required high dose gradients, these are relatively complicated treatment plans.

**Table 1:** Treatment Plans Summary Table 1 shows a summary of the treatment plans. The plans would take longer to deliver than many radiosurgery treatments due to the number of beams and the total MU. However, these plans are within the range of treatment plans that are delivered on a regular basis using the CyberKnife radiosurgery system.

Parameter	Plan 1	Plan 2
Nodes	52	75
Beams	181	192
Max Dose (Gy)	32.05	33.33
Total MU	32001	28651
Min MU	2	10
Max MU	413	396

Table 2 summarizes the doses delivered to target structures for each plan. The volumes of interest (VOI) were defined as follows:

- ‘Box Lesion Set’ Treatment Volume – a structure consisting of a disk through the left atrium anterior to all four pulmonary veins. The outer edge of the disk contains the atrial wall. This excludes the posterior wall target in Plan two.

- PTV – The box volume with a 3 mm isotropic margin.

**Table 2:** Target Dose Summary

	PTV		Box Volume	
	Plan 1	Plan 2	Plan 1	Plan 2
Min Dose (Gy)	18.98	21.93	22.98	23.98
Max Dose (Gy)	31.74	33.33	31.74	33.33
CI	2.02	1.29	5.04	2.52
nCI	2.13	1.33	5.12	2.52
HI	1.28	1.33	1.28	1.33
Coverage (%)	95.03	97.14	98.55	98.84

The columns in Table 2 show the volume of interest (VOI), minimum dose to that volume (min dose), maximum dose to that volume (max dose), conformality index (CI), new conformality index (nCI), homogeneity index (HI) and percentage of the target receiving the prescription dose (coverage). The parameters were calculated by MultiPlan based on methods defined by Accuray.

Table 3 lists the doses delivered to the critical structures that were used for optimization for each plan. Note that the coronary arteries (Circumflex CA and Right CA) and mitral valve annulus (MVA) were not used for optimizing the second plan. Both plans were considered acceptable based on the coverage of the planning target volume (PTV) and the doses to the critical structures.

**Table 3:** Critical Structure Dose Summary

VOI	Plan 1	Plan 2
	Max Dose (Gy)	Max Dose (Gy)
Esophagus	19.01	9.96
Left Bronchus	20.50	14.67
Right Bronchus	18.19	9.01
Circumflex CA	19.90	-
Right CA	14.35	-
MVA	19.71	-

**Figure 1:**
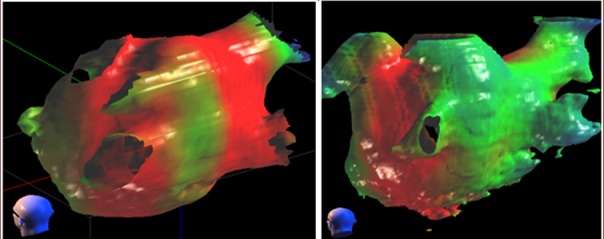
25Gy dose distribution projected on atrial surface The 25 Gy dose distribution in the left atrium for Plan one (left) and Plan two (right) shown as a surface rendering from the left posterior of the patient. The view direction can be seen by the head icon in the lower left part of each panel. The four pulmonary veins can be located as landmarks extending from the posterior of the atrium. The red areas received at least the prescription dose of 25 Gy in each plan while the blue and green areas received less. Plan one used an additional target between the left and right pulmonary vein pairs that were not included in Plan two.

Figure 1 shows the dose distribution in the left atrium at the prescription dose. The use of a hybrid box/WACA (“wide area circumferential ablation” – standard electrophysiology ablation lesion set) lesion set in Plan one produced broad coverage including most of the posterior wall of the atrium. The area nearest the esophagus received a lower dose. For Plan two, no target was defined on the posterior wall of the atrium and this area received a lower dose. From Figure 1, it can be seen that both plans create a wide region that receives more than 25 Gy entirely around the left atrium. Because of the additional target, the left pulmonary veins received substantially more dose in Plan one than in Plan two. Added scarring on the vein ostium would have the therapeutic effect of further blocking aberrant electrical signals.

### Additional contouring

After the plans had been created, additional structures were defined using MultiPlan to determine the dose to the heart. These structures were used for dose calculation only – no re-optimization of the beam set was done based on these new structures. The following structures were created:

- Whole Heart: The cardiac silhouette from the apex to the proximal aortic arch.

- Ventricles: The portion of the whole heart from the valve plane to the apex.

- Heart-Myocardium: The whole heart with the blood pool removed.

- Ventricle-Myocardium: The ventricle structure with the blood pool removed.

- Left Atrium Wall: A 2 mm thick shell around the left atrial blood chamber. The junctions of the pulmonary veins and the mitral valve were removed from this shell.

The contrast enhancement in Plan two was insufficient to allow the coronary arteries to be contoured for dose assessment.

### Results

**Figure 2:**
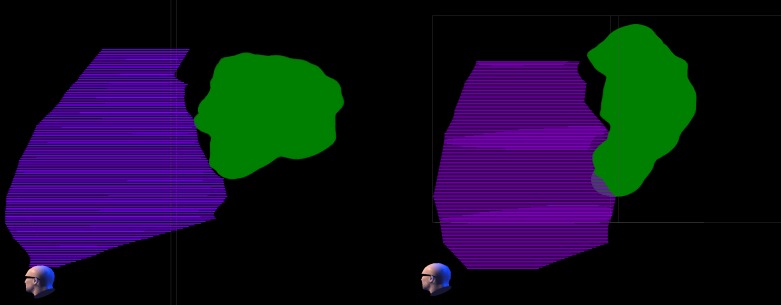
The 25 Gy dose cloud relative to ventricle. The 25 Gy dose cloud (green) is shown relative to the ventricle structure (purple) for Plan one (left) and Plan two (right) in a volume rendering from the left side of the patient. Both left and right ventricles are combined in the ventricle structure. The ventricles are completely spared from the 25 Gy (prescription) dose in Plan one and largely spared in Plan two.

**Figure 3:**
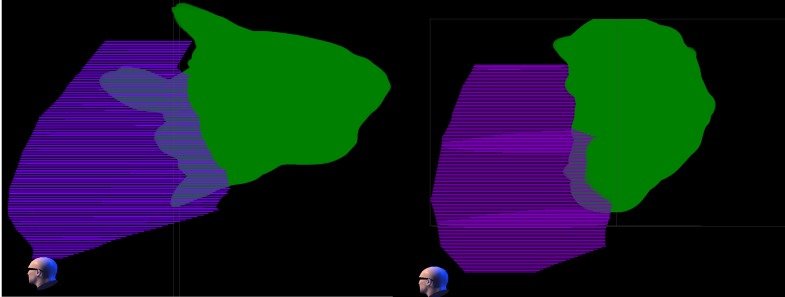
The 16 Gy dose cloud relative to ventricle. The 16 Gy dose cloud (green) is shown relative to the ventricle structure (purple) for Plan one (left) and Plan two (right) in a volume rendering from the left side of the patient.  Both left and right ventricles are combined in the ventricle structure. There is a considerably greater intrusion of the 16 Gy dose into the ventricle in Plan one than in Plan two.

Table 4 lists dose volume results for the additional cardiac volumes in patients one and two. The dose distribution about the additional cardiac structures is shown in Figures 2-3 for the two patients at 25 and 16 Gy.

**Table 4:** Doses to additional heart structures The maximum doses and exposed volumes are listed for each plan/patient under the headings “1” and “2” for each parameter.

Structure	Max Dose (Gy)		Total Vol. (ml)		Vol. at 16 Gy (ml)		Vol. at 18.4 Gy (ml)		Vol. at 22 Gy (ml)		Vol. at 25 Gy (ml)	
Plan	1	2	1	2	1	2	1	2	1	2	1	2
Whole Heart	32.05	33.33	1172	977	253	220	197	182	136	131	92	90
Ventricles	23.98	27.06	619	410	17.8	8.3	4.08	4.23	0.061	0.90	0	0.041
Heart-Myocardium	32.05	32.16	471	391	69.2	48.9	54.8	38.8	36.4	24.8	21.5	13.7
Ventricles-Myocardium	23.98	27.06	244	232	3.6	7.4	1.12	4.04	0.024	0.88	0	0.069
Left Atrial Wall	31.64	32.16	17.6	32.7	15.2	15.6	12.6	13.5	9.95	10.3	6.97	7.1

## Discussion

RTOG 0631 (Radiation Therapy Oncology Group 0631 of the American College of Radiology – Phase II/III Study of Image-Guided Radiosurgery/SBRT for Localized Spine Metastasis) limits the heart volume receiving more than 22 Gy at 0.035 ml and the volume receiving more than 16 Gy at 15 ml for a single fraction spine cancer treatment. Obviously, if the heart is the target, these limits will be violated. However, if only the ventricles are considered as the critical structures, the dose volumes are close to the RTOG guidelines. Table 5 shows a comparison between the RTOG limits and the doses to the ventricles. The ventricular volumes at 16 and 22 Gy in Plan one are very near the RTOG limit but do exceed them. The ventricular myocardial doses, however, are within the guidelines. Plan two, which was optimized without any attempt to limit the dose to the mitral valve annulus, exceeded the 22 Gy RTOG guidelines substantially. 

**Table 5:** Ventricular doses relative to RTOG0631 guidelines

	Plan 1 (ml)	Plan 2 (ml)
RTOG 16 Gy Volume Limit (ml)	16	16
Ventricle at >16 Gy (ml)	17.8	8.3
Ventricle Myocardium at >16 Gy (ml)	3.6	7.4
RTOG 22 Gy Volume Limit (ml)	0.035	0.035
Ventricle at >22 Gy (ml)	0.061	0.90
Ventricle Myocardium at >22 Gy (ml)	0.024	0.88

The randomized international study to compare CyberKnife Stereotactic Radiotherapy with surgical resection in stage I non-small cell lung cancer (STARS) protocol also has limits on the dose to the heart. This study limits the heart volume receiving a total of 35 Gy in four fractions to 10 ml. The biologically effective dose (BED) can be used to compare the effect of one and four fractions. Heart tissue is normal, late-responding tissue so an alpha-beta ratio of 2 Gy is appropriate. With an alpha beta ratio of 2 Gy, a dose of 35 Gy over four fractions is a BED of 188.125 Gy. A single fraction dose of 18.42 Gy would provide this same BED. Table 6 compares the volumes at this dose to the STARS 10ml volume limit. The volume of the ventricle and ventricular myocardium are much smaller than the STARS limit. However, when the atrium is included, the volumes are much higher than the limit.

**Table 6:** Ventricular doses relative to STARS guidelines

	Plan 1 (ml)	Plan 2 (ml)
STARS heart limit: BED > 188.125 Gy (8.74Gy x 4)	10	10
Ventricle at BED > 188.125 Gy (ml)	4.08	4.23
Ventricle Myocardium at BED > 188.125 Gy (ml)	1.12	4.04

The greater ventricular sparing in Plan one is attributed to the use of the mitral valve is dose optimization.

Current guidelines to avoid radiation-induced heart disease are based on large volume exposure of the cardiac silhouette, e.g. treatment for breast cancer, lymphoma, etc. Radiobiology principles do predict a greater tolerance with smaller volume exposure. Although data for fractional organ exposure is non-existent for the heart, it is reasonable to speculate that reducing dose to the ventricles would reduce the risk of coronary artery disease – a common effect of heart exposure in the early Hodgkin’s lymphoma treatment. The sub-anatomy of the heart can be contoured and considered in prospective dose planning. By doing so, a portion of the atrium can be strategically ablated while still approximating the crude RTOG and other dose constraints for the adjacent (non-targeted) heart structures. Future clinical trials in cardiac radiosurgery should accumulate prospective data for the fractional exposure of the heart which would greatly advance the understanding of heart tolerance.

## Conclusions

As shown in this analysis, more than 20% of the cardiac silhouette receives doses greater than 16 Gy during treatments that would isolate the pulmonary veins. However, the dose to the ventricles – where most cardiac adverse events would originate – can be confined to near the dose limits set for heart dose in single fraction spine treatments. The plan that optimized the dose to the mitral valve nearly met the RTOG limits while the plan that did not limit the mitral valve dose had doses to the ventricles that substantially exceeded the limits. It is feasible to create plans that limit the risk of adverse cardiac events by optimizing for the mitral valve dose.
